# Depression proteomic profiling in adolescents with transcriptome analyses in independent cohorts

**DOI:** 10.3389/fpsyt.2024.1372106

**Published:** 2024-05-15

**Authors:** Aleksandr V. Sokolov, Muataz S. Lafta, Didi O. T. Nordberg, Jörgen Jonsson, Helgi B. Schiöth

**Affiliations:** Department of Surgical Sciences, Functional Pharmacology and Neuroscience, Uppsala University, Uppsala, Sweden

**Keywords:** depression, proteome, transcriptome, adolescents, psychiatry

## Abstract

**Introduction:**

Depression is a major global burden with unclear pathophysiology and poor treatment outcomes. Diagnosis of depression continues to rely primarily on behavioral rather than biological methods. Investigating tools that might aid in diagnosing and treating early-onset depression is essential for improving the prognosis of the disease course. While there is increasing evidence of possible biomarkers in adult depression, studies investigating this subject in adolescents are lacking.

**Methods:**

In the current study, we analyzed protein levels in 461 adolescents assessed for depression using the Development and Well-Being Assessment (DAWBA) questionnaire as part of the domestic Psychiatric Health in Adolescent Study conducted in Uppsala, Sweden. We used the Proseek Multiplex Neuro Exploratory panel with Proximity Extension Assay technology provided by Olink Bioscience, followed by transcriptome analyses for the genes corresponding to the significant proteins, using four publicly available cohorts.

**Results:**

We identified a total of seven proteins showing different levels between DAWBA risk groups at nominal significance, including RBKS, CRADD, ASGR1, HMOX2, PPP3R1, CD63, and PMVK. Transcriptomic analyses for these genes showed nominally significant replication of PPP3R1 in two of four cohorts including whole blood and prefrontal cortex, while ASGR1 and CD63 were replicated in only one cohort.

**Discussion:**

Our study on adolescent depression revealed protein-level and transcriptomic differences, particularly in PPP3R1, pointing to the involvement of the calcineurin pathway in depression. Our findings regarding PPP3R1 also support the role of the prefrontal cortex in depression and reinforce the significance of investigating prefrontal cortex-related mechanisms in depression.

## Highlights

- 92 proteins were analyzed in blood from adolescents assessed for depression using DAWBA score.
- Seven proteins showing different levels between DAWBA risk groups were identified.
- Transcriptomic analyses showed nominally significant replication of PPP3R1, ASGR1 and CD63.
- Platelets may be a peripheral key player mirroring the condition of neurons in the CNS.

## Introduction

Depression is a prominent global cause of disability with a lifetime prevalence approaching 20% (1, 2). It can manifest at any stage of life, with the most common period for the first episode typically spanning from adolescence to middle age (1). In its most severe form, depression may lead to suicide attempts, making it one of the leading causes of death among adolescents in Europe (3). It appears to be a multifactorial disease arising from a combination of genetic or metabolic predisposition and environmental factors such as stressful life events and other stressors (4). Currently, the diagnosis of depression relies on clinical assessment, which includes evaluating depressive symptoms like low mood, reduced self-esteem, inappropriate guilt, thoughts of death and suicide, decreased concentration, loss of interest or pleasure in once enjoyable activities, and disturbances in sleep and appetite. Treatment typically involves antidepressant medication, although its efficacy is generally considered mild to moderate (5), with up to 60% of patients experience resistance to treatment (6). Despite major efforts to uncover the pathophysiological mechanisms of depression by analyzing data from genome-wide association studies (7) and RNA gene expression studies (8, 9), our understanding remains limited.

Recent technological advances, particularly within proteomic-based platforms, are providing new insights for better comprehension of depression, going beyond what conventional targeted methods can offer. With the advent of cutting-edge proteomic and transcriptomic techniques allowing simultaneous quantitative assessment of a wide spectrum of proteins or mRNA transcripts, it is now possible to explore not only the functions of individual proteins but also the roles of biochemical pathways and associated metabolites. Proteins serve as the ultimate products of RNA and DNA, often serving as the functional and modifiable units in disease mechanisms. Consequently, proteome analyses hold the potential to elucidate the pathophysiological mechanisms of depression and to identify potential biomarkers for diagnosis, treatment, and monitoring of disease progression. Studies aimed at discovering objective blood-based biomarkers in the fields of psychiatry, neurology, and neuropsychiatry have experienced exponential growth (10–12). However, due to both methodological and clinical challenges associated with psychiatric conditions, such as their inherent complexity and heterogeneous presentation, clinical efficacy remains extremely limited (13–15).

Most studies on risk factors for depression have primarily focused on the adult population. While various biological mechanisms have been explored, no biomarker has been definitively identified or validated for assessing the risk or presence of depression in adolescence and young adulthood (16–20). This is important given that approximately half of depression diagnoses in adults stem from onset in adolescence. Considering the high incidence of depression during the early decades of life and its chronicity throughout life, adolescence presents a unique opportunity to develop effective prevention strategies and alleviate the burden associated with it (21). Furthermore, more research on depression involving youth populations is essential for understanding the natural progression of the disease and identifying factors that may underlie the commonly observed differences in treatment outcomes between adults and children (22). Additionally, developing biomarkers that could predict antidepressant response in the young population would contribute to creating more personalized and effective treatments for young people, a key step in improving the prognosis of depression across the lifespan (23).

In this study, we aimed to analyze protein levels in 461 adolescents assessed for depression as part of the domestic Psychiatric Health in Adolescent Study (PSY cohort) conducted in Uppsala, Sweden, using the Proseek Multiplex Neuro Exploratory panel with Proximity Extension Assay (PEA) technology provided by Olink Bioscience. Subsequently, we conducted transcriptome analyses for the genes corresponding to the significant proteins, using publicly available cohorts, in order to identify differentially expressed transcripts between depressed individuals and controls.

## Materials and methods

### Ethics declarations

This study uses samples from four human sample cohorts other than PSY published prior to the current study. The PSY cohort was conducted in Uppsala, Sweden and approved by the Regional Ethics Committee of Uppsala. All participants gave their written informed consent for participation in the study. Data from the other four cohorts GSE53987, GSE98793, GSE46743, and GSE64930 was deposited in the Gene Expression Omnibus (GEO) portal in the pseudo-anonymized form. The studies deposited in GEO were approved by corresponding regional ethical committees as detailed in the previous publications (24–27).

### PSY subjects

The PSY cohort consists of two phases, including the recruitment phase and the follow-up after approximately one year. The objective of the study was to investigate associations between whole blood biomarkers and different psychiatric phenotypes among school students aged on average between 15-21 years. The phenotypic characterization of the individuals was performed during the visit. Participants self-reported their medications, whereas weight and height were measured. The depressive status of participants was evaluated with a computer-based DAWBA questionnaire (28, 29). The depression band of the questionnaire was used for psychiatric assessment. This band is a numeric score ranging from 0 to 5, where each number corresponds to a probability that an individual has depression: 0 (<0.1%), 1 (~0.5%), 2 (~3%), 3 (~15%), 4 (~50%), and 5 (>70%). We stratified the cohort into high depression risk and low depression risk groups based on the DAWBA score, and individuals with scores 4 and 5 were classified as being depressed. In total, 461 participants successfully completed the DAWBA assessment and had available whole blood and were therefore included in the current study. Among these participants, 334 clearly indicated medications taken, whereas 127 did not fill in the information.

### Proximity extension assay

Protein levels from the 461 participants were assessed using Olink Neuro Exploratory Panel involving 92 proteins (Neuro Exploratory Panel of Olink Proteomics AB, Uppsala Sweden) in two batches. The Neuro Exploratory panel was specifically selected due to the exploratory nature of the current study. This panel was deemed suitable because it offers a broad range of neurological biomarkers, allowing for a proteomic exploration of potential pathways. The Olink methodology has been extensively described in the user manual on the company’s website. Briefly, a mixture of 1 µL of EDTA-containing plasma and a 3-µL incubation mix was incubated overnight at 8°C. Then a 96-μL extension mix with PEA enzyme and PCR reagents was added and incubated at room temperature for 5 min. Afterwards, an extension reaction in a thermal cycler was performed followed by 17 cycles of DNA amplification. During these steps, 92 oligonucleotide-labeled pairs of antibodies were allowed to bind to their respective target protein in the sample. Once the antibody probes bound to the targeted protein and the attached DNA oligonucleotides were in close proximity, the oligonucleotides hybridized and were extended by enzymatic polymerization. The oligonucleotide templates were then amplified and quantified using real-time polymerase chain reaction. Protein levels are expressed as Normalized Protein Expression (NPX) values on log2-scale (30). The assay procedures were performed at Affinity Proteomics Uppsala SciLifeLab in Sweden.

### Transcriptome validation cohorts

The validation of the results from the PSY cohort was performed utilizing a publicly-available transcriptome cohort with post-mortem brain tissues (GSE53987) and three cohorts with the whole blood (GSE98793, GSE46743, and GSE64930). The cohort GSE53987 comprises post-mortem brain sample data for major depressive disorder (MDD), schizophrenia, bipolar disorder, and controls. Descriptions of sample collection and preparation are provided in the original publication (27). Transcriptome data from three brain tissues was available and included the associative striatum, hippocampus, and prefrontal cortex (Brodmann area 46) for each of the disorders and controls. We compared transcriptome profiles of MDD patients (n = 16 – 17) versus controls (n = 18 – 19) separately for each of the three brain tissues.

The cohort GSE98793 included 192 participants and is a part of GlaxoSmithKline–High-Throughput Disease-specific target Identification Program. The sample includes 64 individuals with MDD, 64 participants with comorbid MDD and anxiety, and 64 healthy controls. Dataset includes covariables on age, sex, diagnoses (anxiety and MDD), as well as a batch covariable. Please refer to the initial publication for detailed descriptions (26).

The cohort GSE46743 is a part of a large-scale study conducted at the Max Planck Institute of Psychiatry, Munich, Germany. The study objective was to investigate transcriptome reaction to stress in the context of depression. The study investigated whole blood transcriptome profiles before and after exposure to dexamethasone and involved 160 male subjects. Data on age and BMI was collected during the study. Transcriptome profiling was performed with Illumina HumanHT-12 expression beadchip. Further information could be found elsewhere (24). In the present work, we used transcriptome profiles before exposure to dexamethasone to compare the baseline expression in depression patients versus controls.

Lastly, the cohort GSE64930 had a similar design to GSE46743 and investigated transcriptome reaction to stress in the context of depression. This cohort has a partial overlap consisting of 79 participants with GSE46743. However, it included both female and male participants (n=289) (25). The initial deposited phenotypic data contains information only on participant’s sex. Data on age, BMI, RNA integrity number, and Hamilton Depression Rating Scale (HAM-D) scores, as well as three surrogate variables to adjust for cell heterogeneity, was provided by the study investigator after a request. Similar to GSE46743, we used the data before dexamethasone exposure to compare expression in depressed participants versus controls. In total, 286 participants were amenable for analysis as three samples did not have information on RNA integrity and were excluded.

### Data preprocessing

Transcriptome data preparation and analysis were performed in the R programming language environment (version 4.2.0). Data for GSE98793 and GSE53987 was available in the form of raw CEL files. The R package *affy* was used to perform background correction, normalization, and summarization (31). The initial array images were inspected for potential visual artifacts. The initial data had substantial variation due to batching. Data preparation was performed in the expresso framework (*affy*), where all data curation steps are done sequentially. The background was corrected with a robust multi-array average method. The resulting log2-transformed values were quantile normalized and probe-wise correction was based exclusively on the perfect match intensities (“pmonly”). The resulting expression values were obtained with the “medianpolish” procedure. The final correction for batching was performed with the function *ComBat* from the R package *sva* (32). The batch correction was performed with covariates and included subject group, anxiety status, gender, and age to preserve corresponding biological effects.

In the cohorts GSE46743 and GSE64930, we used already preprocessed filtered data. Expression values corrected for background that passed through variance stabilization and normalization (VSN) procedure were downloaded directly from the GEO from the corresponding records.

Data for the PSY cohort comes in the form of analyzable normalized NPX values. We performed additional correction for the batch effect (plate) using the function *ComBat* from the R package *sva*. The batch correction was performed with covariates that included DAWBA risk group, age, sex, as well as the antidepressant intake (binary). The antidepressant covariate was only included for the samples subject to differential expression analysis adjusted for antidepressant intake.

### Statistical analysis and data visualization

Linear regression models (R implementation) were used to investigate associations between protein/transcript expression and the depression status in all cohorts. The choice of model covariates was based on the available data and biological relevance. In each model, the level of protein/transcript was regressed against the depression status or DAWBA risk group (binary) adjusted for covariates. In the PSY cohort, the covariates included age (numeric) and sex (binary). In the analysis adjusted for antidepressant intake, the antidepressant covariate (binary) was added. The analysis adjusted for antidepressant intake was performed as a “complete case” and all participants that did not report the data on antidepressant intake were excluded. In the GSE53987 cohort, the covariates were sex (binary), ethnic background (binary), age (numeric), tissue pH (numeric), post-mortem interval (numeric), and RNA integrity number (numeric). In the GSE53987 cohort, the covariates included anxiety comorbidity (binary), sex (binary), age (numeric). In the GSE46743 cohort, the only covariate included was age (numeric) as all participants were male. Lastly, sex (binary), age (numeric), RNA integrity number (numeric), and three surrogate variables (numeric) were used as covariates in GSE64930 cohort. A two-tailed nominal p < 0.05 was considered significant and only results reproduced in at least one cohort were deemed as relevant. The Bonferroni-adjusted p-values were calculated along the nominal statistics. Data was visualized using custom R scripts with R packages *ggplot2* and *visNetwork*.

## Results

### Demographic data

The demographic details of the participants in the PSY cohort are presented in **Table 1**. The initial dataset included 461 participants, of whom 138 had available proteomic data from recruitment, and 353 had proteomic data from the follow-up. After conducting quality control steps in connection with the proteomic analysis, five samples from the screening and four samples from the recall had quality control warnings and were subsequently removed from the analyses. Out of the 461 participants, 61 were in the high-risk DAWBA group, of which the majority (91.8%) were females. The age was similar in both the high and low-risk DAWBA groups (mean age 16.67 vs. 17.26 years). While five participants in the high-risk DAWBA group reported taking antidepressants, only nine participants reported taking antidepressants in the low-risk DAWBA group. Thus, 121 participants (> 30%) did not answer the drug intake question in the low-risk DAWBA group.

**Table 1:** PSY cohort.

PSY		
Initial dataset includes 461 participants (138 from the recruitment and 353 the follow-up)		
Participants with missing data excluded: 0 (for the base model*) Participants with missing data excluded: 127 (adjusted for antidepressants) Resulting number of participants: 461 (base model) Resulting number of participants: 334 (adjusted for antidepressants)		
**DAWBA risk group**	Control: 400 (100%) Depressed: 0 (0%)	Control: 0 (0%) Depressed: 61 (100%)
**Gender**	Male: 104 (26%) Female: 296 (74%)	Male: 5 (8.2%) Female: 56 (91.8%)
**Age**	17.26 ± 1.27 Min: 15, Max: 21	16.67 ± 1.35 Min: 15, Max: 20
**Antidepressants**	No: 270 (67.5%) Yes: 9 (2.2%) Not reported: 121 (30.2%)	No: 50 (82%) Yes: 5 (8.2%) Not reported: 6 (9.8%)

This table provides demographic characteristics for the PSY cohort. Base model* indicates the analysis non-adjusted for antidepressant intake. The analysis with antidepressants was performed as a “complete case” and all participants that did not report data on antidepressant intake were excluded. Categorical variables are shown as counts with their associated percent (in relation to a subgroup). Numerical variables are shown as mean ± standard deviation, as well as minimal and maximal values below. DAWBA, Development and Well-Being Assessment.

The demographic details of the participants in each of the four publicly available transcriptome validation cohorts are presented in **Supplementary Tables S1–4**. Each cohort was stratified into two groups based on depression diagnosis, depressed or non-depressed (see methods). The cohort GSE53987 was nearly identical in all characteristics between the depressed and non-depressed groups (**Supplementary Table S1**). In GSE98793, there was no difference between groups except for a relatively higher proportion of females than males (75% vs. 25%) in both the depressed and non-depressed groups (**Supplementary Table S2**). In the GSE46743 cohort, the mean age of participants was relatively higher in the depressed group (48.39 vs. 40.18) than in the non-depressed group (**Supplementary Table S3**). Lastly, for the GSE64930 cohort, there was a higher proportion of males than females (64.3% vs. 35.7%) in the depressed group and (71.9% vs. 28.1%) in the non-depressed group, while the mean age was higher in the depressed group (48.06 vs. 38.55) than in the non-depressed group (**Supplementary Table S4**).

### Differential expression (proteome) analyses in PSY cohort

**Figure 1:**
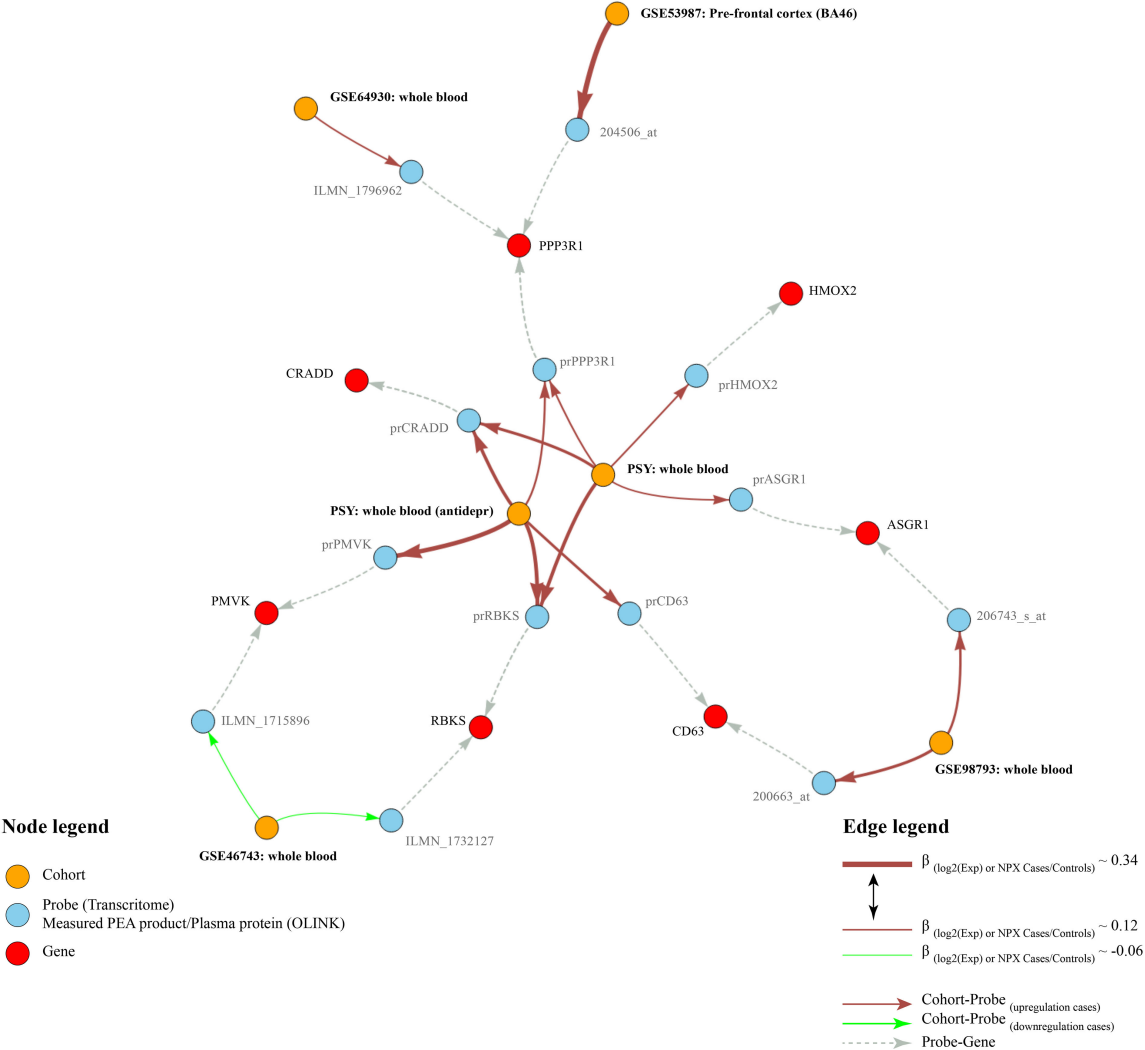
Results graph. This figure shows the main results obtained in the PSY cohort and their replications in the transcriptome cohorts. Red nodes indicate differentially expressed genes, blue nodes indicate probes or plasma proteins, orange nodes indicate the analyses. Arrows indicate relationships between the nodes. Brick-red edges indicate upregulation of the protein/probe in depression, whereas green edges indicate respective downregulation. Gray edges show relationships between gene names and probes. The thickness of the red and green edges edges is proportional to the sizes of *β*-coefficients in the linear models.

Out of the 92 protein biomarkers measured using the Proseek Multiplex Neuro Exploratory panel, only 43 proteins were detected in more than 75% of blood samples. Therefore, 49 proteins were excluded from the analysis. We conducted linear regression models, adjusted for age and sex, and both with and without adjustment for antidepressants, to investigate the differences in protein levels between high-risk and low-risk DAWBA participants. Five proteins (RBKS, CRADD, ASGR1, HMOX2, and PPP3R1) showed different levels between DAWBA risk groups at a nominal significance in the linear models non-adjusted for antidepressants (**Supplementary Table S5**). After adjustment for antidepressants, only three of these proteins (RBKS, CRADD, and PPP3R1) showed nominally significant differences in levels (**Supplementary Table S6**). The estimated effect sizes of proteins in the depressant-adjusted analysis were slightly larger than in the non-adjusted models for antidepressant intake. A summary of the main results obtained in the PSY cohort and their replications in the transcriptome cohorts is shown in **Figure 1**.

### Transcriptome analyses in independent validation cohorts

**Figure 2:**
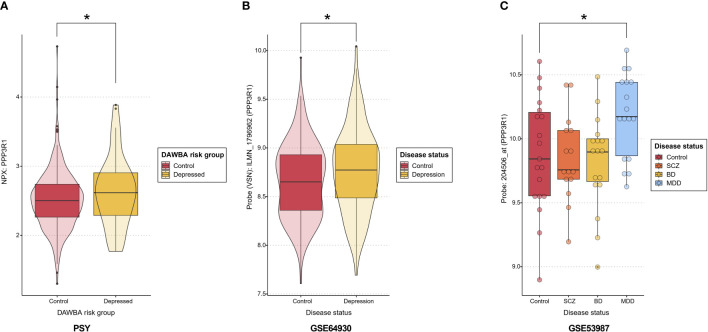
PPP3R1 expression. **(A)** PPP3R1 plasma NPX in the PSY cohort depending on the DAWBA risk group. Asterisk indicates a nominal p<0.05. **(B)** The expression levels of PPP3R1-related probe ILMN_1796962 in whole blood in depression versus controls in GSE64930. Asterisk indicates a nominal p<0.05. **(C)** The expression levels of PPP3R1-related probe 204506_at in prefrontal cortex in several psychiatric conditions and controls in the dataset GSE53987. Asterisk indicates a nominal p<0.05. DAWBA, Development and Well-Being Assessment; NPX, Normalized Protein Expression; SCZ, schizophrenia; BD, bipolar disorder; MDD, major depressive disorder.

Transcriptome analyses were conducted for the genes corresponding to the nominally significant proteins using four publicly available cohorts to investigate differentially expressed transcripts. Our differential expression analyses of gene-related probes showed nominal upregulation in depression for PPP3R1, which was replicated in both GSE53987 (prefrontal cortex) (**Supplementary Table S7**) and GSE64930 (whole blood) (**Supplementary Table S8**), consistent with the upregulation observed in the PSY cohort. No transcripts were differentially expressed in the hippocampus or associative striatum in GSE53987 (**Supplementary Table S7**). A summary of the expression levels of PPP3R1 at the proteomic level in the PSY cohort and the transcriptomic level in GSE53987 and GSE64930 is shown in **Figure 2**, while **Supplementary Figure S1** displays PPP3R1 expression across cohorts for better visualization of effect sizes. Moreover, we also observed nominal significance for ASGR1 and Bonferroni-adjusted significance for CD63, both of which were upregulated in depressed cases in GSE98793, aligning with the direction of upregulation observed in the PSY cohort (**Supplementary Table S9**). Lastly, PMVK and RBKS were nominally significant in GSE46743, but the directions did not match with the PSY cohort (**Supplementary Table S10**).

## Discussion

In this exploratory study, the Olink Neuro Exploratory panel was used to analyze the expression levels of 92 proteins among adolescents based on their DAWBA risk in the PSY cohort, followed by transcriptome analyses in independent cohorts. As a result, we identified a total of seven proteins showing different levels between DAWBA risk groups at nominal significance, whether adjusted or non-adjusted for antidepressant use, including RBKS, CRADD, ASGR1, HMOX2, PPP3R1, CD63, PMVK. Transcriptomic analyses for these genes showed nominally significant replication of PPP3R1 in two of four cohorts, while ASGR1 and CD63 were replicated in only one cohort.

One protein that drew particular attention due to its differential expression is PPP3R1, as it showed nominal significance both before and after adjustment for antidepressant use, and it was the only protein with consistent directions of change in two transcriptome cohorts. PPP3R1 is a 19 kDa calcium-binding regulatory subunit of the calcium-dependent protein phosphatase, also known as calcineurin. Abundantly expressed in human brain neuronal cell cytosol, PPP3R1 plays a critical role in the calcineurin signaling pathway (33, 34). Studies have observed that mice with a PPP3R1 mutation, eliminating calcineurin activity in somatic cells, exhibited structural defects in anterior neural structures (35). There is increasing evidence suggesting that calcineurin signaling is implicated in the pathophysiology of depression and its treatment. The association between calcineurin activity and psychiatric disorders is based on clinical observations that the rates of anxiety and depression increase in patients treated chronically with the calcineurin inhibitor cyclosporine-A to prevent rejection after organ transplantation (36). Preclinical studies reveal depressive-like behavior induction in mice through amygdala calcineurin inhibition (37). Additionally, chronic stress in rats, a model for depression research, reduces calcineurin activity in the hippocampal CA3 region (38). Notably, calcineurin has also been found to have direct antidepressant-like effects, as the inhibition of calcineurin in the prefrontal cortex of rats induces depressive-like behavior (39). These findings taken together support the role of calcineurin in the pathophysiology of depression.

PPP3R1 was replicated in two transcriptomic cohorts, GSE53987 [prefrontal cortex (PFC)] and GSE64930 (whole blood). The differential expression of PPP3R1 in the prefrontal cortex of depressed adolescents aligns with the existing evidence that the PFC is one of the regions most consistently affected in depression. Extensive literature has strongly implicated PFC dysfunction in MDD, revealing functional, structural, and systems-level abnormalities spanning various PFC regions (40). Evidence of structural changes in the PFC associated with depression mainly comes from secondary measures. For instance, the volume of the PFC is reduced in depressed patients, and this decrease is correlated with the duration of illness (41). In depression, specific changes can be observed in the molecular composition of PFC neurons (42), which modulate their electrical properties (43). Decreases in the volume of the orbitofrontal cortex (44) and pyramidal cell density (45) have also been observed in depression. These studies suggest that PFC neurons undergo widespread physiological and morphological alterations during depression. Other lines of evidence include dysregulation of glutamatergic and GABAergic neurotransmission in the PFC. PFC glutamate metabolites are reduced in depression (46), and postmortem studies demonstrate changes in ionotropic and metabotropic glutamate receptors (47, 48). Medial PFC levels of the GABA synthetic enzyme glutamate decarboxylase-67 are also reduced in postmortem brains of depressed subjects (30), as are markers of the somatostatin/calbindin (SST) GABAergic subtype (49, 50).

Other interesting findings involve two proteins, ASGR1 and CD63, corresponding to genes that may offer new insights into the pathophysiological processes of depression. Both proteins were replicated with consistent changes, but only in one cohort, GSE98793, where CD63 remained significant after Bonferroni correction. CD63 belongs to the tetraspanin family and is characterized by its four transmembrane domains (51). It can associate with interaction partners, including integrins, receptors, kinases, and other tetraspanin proteins, on the cell surface, thus contributing to the regulation of multiple signaling pathways (51–54). CD63 also serves as a platelet dense granule and lysosomal membrane protein. Interestingly, CD63 exhibits high expression in patients with comorbid diabetes and depression compared to those with diabetes alone. This comorbidity results in enhanced platelet hyperactivation and a pro-inflammatory state, increasing susceptibility to vascular complications (55). On the other hand, ASGR1 is the major subunit of the asialoglycoprotein receptor (ASGPR), a liver-specific lectin that plays a role in glycoprotein homeostasis (56). Variants in ASGR1 are associated with lower non-high-density lipoprotein (non-HDL) cholesterol levels and a reduced risk of coronary artery disease (57, 58). Thus, ASGR1 holds clinical potential as a target for lowering blood cholesterol levels. However, there is limited information available regarding the regional or cellular specificity of ASGR1 in the human brain, and its function in the brain remains poorly understood (59).

**Figure 3:**
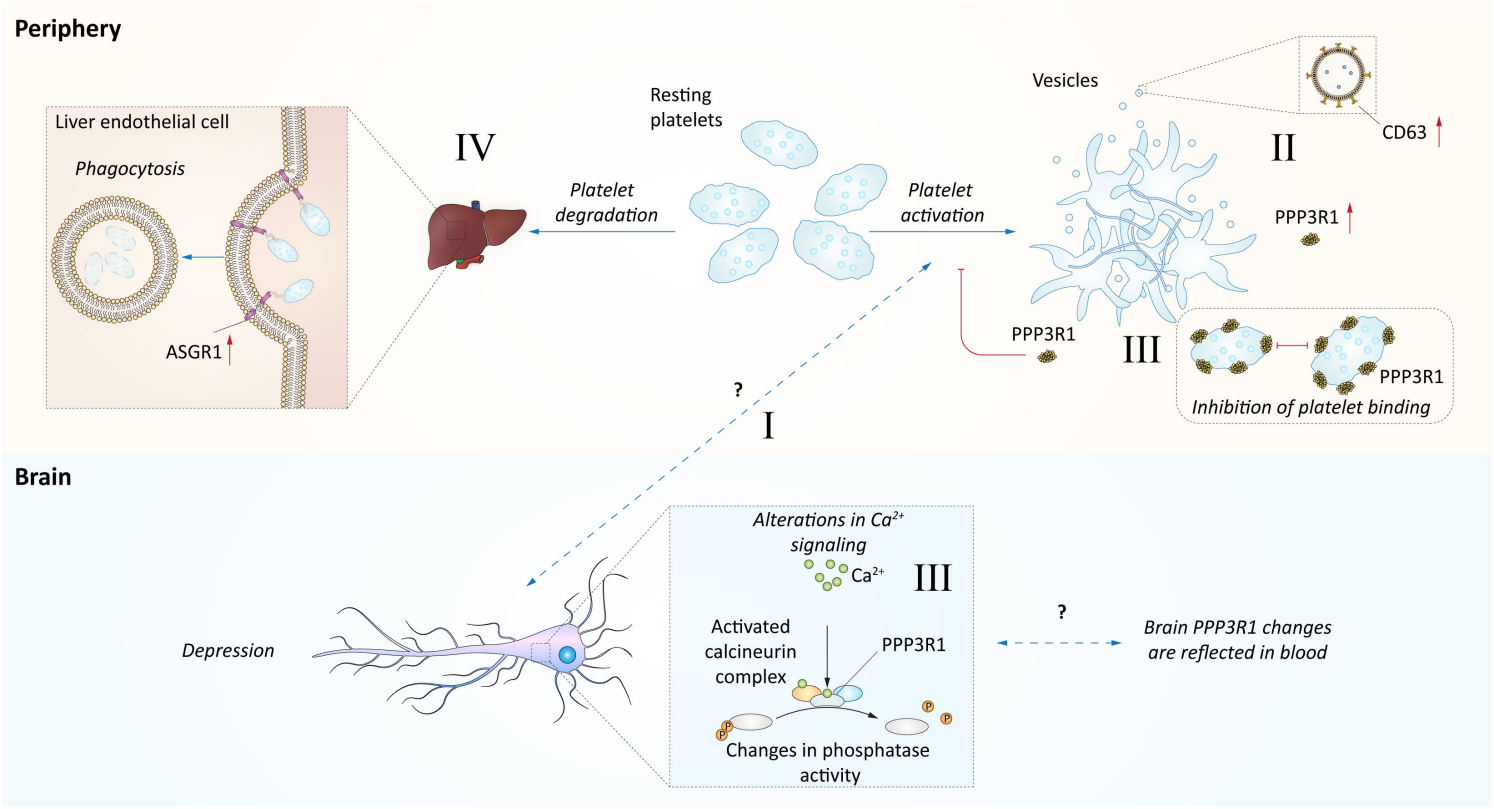
Hypothetical model of blood proteome relation with depression. This figure represents a hypothetical model explaining observed associations between depression and the blood levels of PPP3R1, CD63, and ASGR1. I Several studies indicate links between platelet activation and depression (60, 61). However, this mechanism is not fully established. II. The activation of platelets could be indirectly indicated by increase of CD63 levels, as this protein is abundant in exosomes (62) and release of exosomes could be attributed to platelet activation (63). The levels of CD63 platelets were positively associated with depression (60, 61). III. PPP3R1, a regulatory component of calcineurin complex mediating phosphatase activity, has been shown to bind platelets and prevent their aggregation (64, 65), and thus its increase may indicate disturbances in platelet homeostasis. The disturbances of calcineurin signaling may be indicated by changes in blood and prefrontal cortex as observed in this study. However, we observed increased plasma levels of PPP3R1 in depressed individuals. IV. The ASGR1 contribution to depression is not clear, however it is related to platelet phagocytosis by liver sinusoidal endothelial cells (66, 67).

In the search of an explanatory model for the observed associations between depression and the blood levels of PPP3R1, CD63, and ASGR1, platelets emerge as potentially pivotal players (**Figure 3**). The presence of free CD63 in plasma strongly suggests its exosomal origin, as CD63 is widely recognized as one of the most specific exosomal markers (62). Notably, activated blood platelets are a major source of exosomes in human blood (63). This finding raises the possibility of a positive association between activated blood platelets and depression, a notion supported by multiple studies linking platelet activation to depression (60, 61). Additionally, it’s worth noting that ASGR1 plays a vital role in platelet clearance by the liver, both in humans and pigs (66, 67). Moreover, genetic variants of ASGR1 has been reported to influence platelet activation (68). Furthermore, PPP3R1, a regulatory component of the calcineurin complex responsible for phosphatase activity, has been observed to bind to platelets and prevent their aggregation (64, 65). Consequently, an increase in PPP3R1 levels may signify disruptions in platelet homeostasis.

In summary, while this model may involve some speculation, it is reasonable to suspect that the identification of three proteins related to platelet activation status in depressed adolescents could potentially reflect the neuropathological status of these adolescents. This notion is supported by a substantial body of scientific evidence suggesting that blood platelets may mirror the condition of neurons in the central nervous system (CNS) (69–74). To highlight some specific roles of platelets, studies of the serotonin uptake, storage, and secretion mechanisms in platelets have indicated major similarities to neurons (75). Studies investigating the comorbidity between cardiovascular disease and depression have proposed platelet serotonin signaling as a potential mechanism for the higher incidence of cardiac adverse events in depressed cardiovascular patients (76). Additionally, other studies have suggested that altered platelet reactivity might link stress, depression, and cardiovascular disease through changes in platelet aggregability and their content of bioactive compounds (77, 78). Platelet activation has also been implicated as a contributor to the progression of neurological conditions such as Alzheimer’s disease (79). There is potential for platelet interactions with systemic factors that change concurrently with CNS alterations, underscoring the importance of future studies elucidating the numerous systemic factors that change during depression onset, e.g. in adolescence, and how they interact with platelets.

The study’s results should be interpreted in light of several limitations. Firstly, our proteomic platform covers a rather selective portion of the proteome, potentially missing numerous proteomic pathways related to depression that our methods did not capture. Additionally, a significant number of proteins in the selected panel appear to be unexpressed in whole blood, as only 43 out of 92 were analyzable. This limitation might be attributed to the use of peripheral fluid biomarkers, as concentrations measured in the periphery may not necessarily reflect pathophysiological processes in the CNS. Furthermore, depression is a heterogeneous group of diseases often occurring concurrently with or induced by other conditions. Consequently, it is challenging to select a molecularly homogeneous group of patients or samples.

Additional limitations include the nominal significance observed for almost all proteins, which may provide weak evidence. However, it’s worth noting that nominal replication at the transcriptome level with consistent directions in independent cohorts could be considered stronger evidence. Another limitation is related to the characterization of depression across all cohorts, which is not consistent and may not represent the same phenotype. Furthermore, the populations in the cohorts are diverse and not easily comparable. Despite our efforts to account for some of the differences between the cohorts, such as age, there is still a lack of age overlap between the PSY cohort and the transcriptome cohorts. This necessitates cautious interpretation of the discordant results between the cohorts. However, on the other hand, the current findings in the PSY cohort, which reveal changes associated with depression that differ from those observed in older patients in the transcriptomic cohorts, may offer valuable insights into the etiology of depression onset during adolescence. Lastly, there were many missing values regarding medication in the PSY cohort. Since antidepressant treatment can be a significant confounder, we conducted analyses both with and without adjustment for antidepressants. Adjusting for antidepressants led to a smaller sample size due to the exclusion of missing values. We observed slightly increased effect size in the adjusted analysis that should be interpreted carefully. It could be related to biological effects of antidepressants masking protein effect in non-adjusted models or it may be of random origin as the accuracy of estimates is smaller in the adjusted analysis. Consistently, the observed shifts in the estimates of matching proteins matched between analyses (PPP3R1, RBKS, and CRADD) were less than corresponding standard errors. Lastly, even though the analysis was adjusted for antidepressants, it is possible that the real prevalence of antidepressant use may be underestimated due to underreporting from study participants. However, our estimated prevalence of antidepressant used in the PSY cohort of ~4.2% is comparable to the recent report on antidepressant use in Sweden of ~4.76 (age group 10-19 years) (80).

## Conclusion

Our study of adolescent depression revealed protein-level and transcriptomic differences, particularly in PPP3R1, pointing to the involvement of the calcineurin pathway in depression. Our finding with regard to PPP3R1 also support the role of the prefrontal cortex in depression and reinforces the significance of investigating PFC-related mechanisms in depression. However, it’s important to consider these findings within the context of certain limitations, such as the selectivity of our proteomic platform and variations in depression characterization across cohorts. Further research is needed to validate and expand upon these findings, potentially leading to improved diagnostic and therapeutic strategies for adolescent depression.

## Data availability statement

The original contributions presented in the study are included in the article/**Supplementary Material**. Further inquiries can be directed to the corresponding author.

## Ethics statement

The studies involving humans were approved by Regional Ethics Committee of Uppsala, Sweden. The studies were conducted in accordance with the local legislation and institutional requirements. Written informed consent for participation in this study was provided by the participants’ legal guardians/next of kin.

## Author contributions

AS: Conceptualization, Data curation, Formal analysis, Methodology, Visualization, Writing – review & editing. ML: Conceptualization, Formal analysis, Methodology, Writing – original draft. DN: Methodology, Writing – review & editing. JJ: Conceptualization, Writing – review & editing. HS: Conceptualization, Funding acquisition, Supervision, Writing – review & editing.

## Supplementary material

Supplementary Figure 1 PPP3R1 expression. This figure shows the PPP3R1 expression across cohorts in the form of bars for better effect size visualization. **(A)** PPP3R1 plasma NPX in the PSY cohort depending on the DAWBA risk group. Asterisk indicates a nominal p<0.05. Error bar shows the standard error of the mean. **(B)** The expression levels of PPP3R1-related probe ILMN_1796962 in whole blood in depression versus controls in GSE64930. Asterisk indicates a nominal p<0.05. Error bar shows the standard error of the mean. **(C)** The expression levels of PPP3R1-related probe 204506_at in prefrontal cortex in several psychiatric conditions and controls in the dataset GSE53987. Error bar shows the standard error of the mean. Asterisk indicates a nominal p<0.05. DAWBA, Development and Well-Being Assessment; NPX, Normalized Protein Expression (NPX); SCZ, schizophrenia; BD, bipolar disorder; MDD, major depressive disorder.
