# Supplementary figures and images for: Depression proteomic profiling in adolescents with transcriptome analyses in independent cohorts
Source: Front Psychiatry. 2024 May 15;15:1372106. doi: 10.3389/fpsyt.2024.1372106 (PMC11133714; doi:10.3389/fpsyt.2024.1372106)

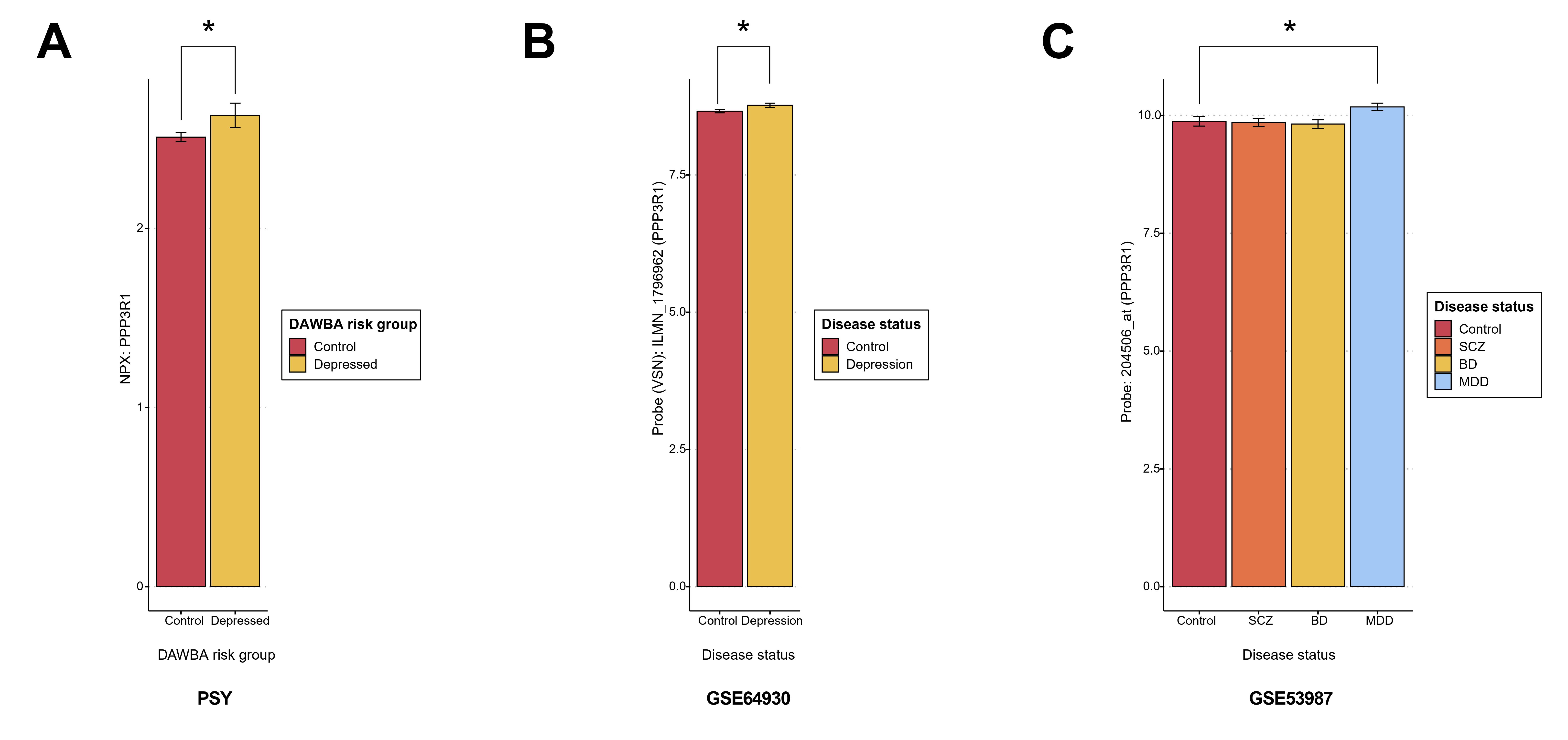

Supplement: Supplementary Figure 1 — PPP3R1 expression. This figure shows the PPP3R1 expression across cohorts in the form of bars for better effect size visualization. (A) PPP3R1 plasma NPX in the PSY cohort depending on the DAWBA risk group. Asterisk indicates a nominal p<0.05. Error bar shows the standard error of the mean. (B) The expression levels of PPP3R1-related probe ILMN_1796962 in whole blood in depression versus controls in GSE64930. Asterisk indicates a nominal p<0.05. Error bar shows the standard error of the mean. (C) The expression levels of PPP3R1-related probe 204506_at in prefrontal cortex in several psychiatric conditions and controls in the dataset GSE53987. Error bar shows the standard error of the mean. Asterisk indicates a nominal p<0.05. DAWBA, Development and Well-Being Assessment; NPX, Normalized Protein Expression (NPX); SCZ, schizophrenia; BD, bipolar disorder; MDD, major depressive disorder. [file Image_1.jpeg]
