# Supplementary material for: Depression proteomic profiling in adolescents with transcriptome analyses in independent cohorts
Source: Front Psychiatry. 2024 May 15;15:1372106. doi: 10.3389/fpsyt.2024.1372106 (PMC11133714; doi:10.3389/fpsyt.2024.1372106)
Supplement: Supplementary file 2 [file DataSheet_1.docx]

**Supplementary tables**

**Table S1 GSE53987**

| **GSE53987: hippocampus** | | |
| --- | --- | --- |
| Initial dataset includes 35 participants | | |
| Participants with missing data excluded: 0  Resulting number of participants: 35 | | |
| **Diagnosis** | MDD: 17 (100%)  Healthy: 0 (0%) | MDD: 0 (0%)  Healthy: 18 (100%) |
| **Gender** | Female: 8 (47.1%)  Male: 9 (52.9%) | Female: 9 (50%)  Male: 9 (50%) |
| **Ethnic background** | White: 16 (94.1%)  Black: 1 (5.9%) | White: 17 (94.4%)  Black: 1 (5.6%) |
| **Age** | 45.18 ± 10.71  Min: 26, Max: 62 | 48.17 ± 10.95  Min: 22, Max: 68 |
| **Tissue pH** | 6.58 ± 0.24  Min: 6.2, Max: 7.3 | 6.61 ± 0.21  Min: 6.1, Max: 7.1 |
| **RIN** | 7.06 ± 0.87  Min: 5.5, Max: 8.4 | 7.37 ± 0.64  Min: 6.1, Max: 8.5 |
| **GSE53987: Pre-frontal cortex (BA46)** | | |
| Initial dataset includes 36 participants | | |
| Participants with missing data excluded: 0  Resulting number of participants: 36 | | |
| **Diagnosis** | MDD: 17 (100%)  Healthy: 0 (0%) | MDD: 0 (0%)  Healthy: 19 (100%) |
| **Gender** | Female: 8 (47.1%)  Male: 9 (52.9%) | Female: 9 (47.4%)  Male: 10 (52.6%) |
| **Ethnic background** | White: 16 (94.1%)  Black: 1 (5.9%) | White: 18 (94.7%)  Black: 1 (5.3%) |
| **Age** | 45.18 ± 10.71  Min: 26, Max: 62 | 48.05 ± 10.65  Min: 22, Max: 68 |
| **Tissue pH** | 6.58 ± 0.24  Min: 6.2, Max: 7.3 | 6.59 ± 0.22  Min: 6.1, Max: 7.1 |
| **RIN** | 7.75 ± 0.5  Min: 6.7, Max: 8.4 | 7.85 ± 0.62  Min: 6.6, Max: 8.7 |
| **GSE53987: Associative striatum** | | |
| Initial dataset includes 34 participants | | |
| Participants with missing data excluded: 0  Resulting number of participants: 34 | | |
| **Diagnosis** | MDD: 16 (100%)  Healthy: 0 (0%) | MDD: 0 (0%)  Healthy: 18 (100%) |
| **Gender** | Female: 6 (37.5%)  Male: 10 (62.5%) | Female: 8 (44.4%)  Male: 10 (55.6%) |
| **Ethnic background** | White: 16 (100%)  Black: 0 (0%) | White: 17 (94.4%)  Black: 1 (5.6%) |
| **Age** | 46.5 ± 9.96  Min: 26, Max: 62 | 48.44 ± 10.82  Min: 22, Max: 68 |
| **Tissue.pH** | 6.58 ± 0.25  Min: 6.2, Max: 7.3 | 6.59 ± 0.23  Min: 6.1, Max: 7.1 |
| **RIN** | 8.24 ± 0.83  Min: 6.7, Max: 9.2 | 8.21 ± 0.69  Min: 7, Max: 9.3 |

**Table S1.** This table shows demographic characteristics of the cohort GSE53987 in three different tissues. Categorical variables are shown as counts and their associated percent (in relation to a subgroup). Numerical variables are shown as mean ± standard deviation, as well as minimal and maximal values below. Abbreviations: MDD, major depressive disorder; RIN, RNA integrity number.

**Table S2 GSE98793**

| **GSE98793** | | |
| --- | --- | --- |
| Initial dataset includes 192 participants | | |
| Participants with missing data excluded: 0  Resulting number of participants: 192 | | |
| **Diagnosis** | Healthy: 64 (100%)  MDD: 0 (0%) | Healthy: 0 (0%)  MDD: 128 (100%) |
| **Gender** | Female: 48 (75%)  Male: 16 (25%) | Female: 96 (75%)  Male: 32 (25%) |
| **Age** | 52.03 ± 11.41  Min: 31.2, Max: 73.1 | 52.04 ± 11.51  Min: 31, Max: 72.8 |
| **Anxiety** | Yes: 0 (0%)  No: 64 (100%) | Yes: 64 (50%)  No: 64 (50%) |

**Table S2.** This table shows demographic characteristics of the cohort GSE98793. Categorical variables are shown as counts and their associated percent (in relation to a subgroup). Numerical variables are shown as mean ± standard deviation, as well as minimal and maximal values below. Abbreviations: MDD, major depressive disorder.

**Table S3 GSE46743**

| **GSE46743** | | |
| --- | --- | --- |
| Initial dataset includes 160 participants | | |
| Participants with missing data excluded: 0  Resulting number of participants: 160 | | |
| **Diagnosis** | Healthy: 91 (100%)  MDD: 0 (0%) | Healthy: 0 (0%)  MDD: 69 (100%) |
| **Age** | 40.18 ± 12.45  Min: 18, Max: 61 | 48.39 ± 13.59  Min: 21, Max: 73 |

**Table S3.** This table shows demographic characteristics of the cohort GSE46743. Categorical variables are shown as counts and their associated percent (in relation to a subgroup). Numerical variables are shown as mean ± standard deviation, as well as minimal and maximal values below. All participants in the cohort were males. Abbreviations: MDD, major depressive disorder.

**Table S4 GSE64930**

| GSE64930 | | |
| --- | --- | --- |
| Initial dataset includes 289 participants | | |
| Participants with missing data excluded: 3  Resulting number of participants: 286 | | |
| Status | Healthy: 160 (100%)  MDD: 0 (0%) | Healthy: 0 (0%)  MDD: 126 (100%) |
| Sex | Female: 45 (28.1%)  Male: 115 (71.9%) | Female: 45 (35.7%)  Male: 81 (64.3%) |
| Age | 38.55 ± 12.27  Min: 18, Max: 62 | 48.06 ± 14.23  Min: 21, Max: 75 |
| RIN | 7.96 ± 0.59  Min: 4.5, Max: 9 | 8.14 ± 0.46  Min: 6.5, Max: 9.2 |
| HAMD | 0.6 ± 1.17  Missing val: 9 (5.62%)  Min: 0, Max: 6 | 23.79 ± 7.5  Missing val: 22 (17.46%)  Min: 8, Max: 38 |

**Table S4.** This table shows demographic characteristics of the cohort GSE64930. Categorical variables are shown as counts and their associated percent (in relation to a subgroup). Numerical variables are shown as mean ± standard deviation, as well as minimal and maximal values below. All participants in the cohort were males. Abbreviations: MDD, major depressive disorder; RIN, RNA integrity number; HAMD, Hamilton Depression Rating Scale.

**Descriptions for Tables S5-S10**

**Table S5.** This table shows results for differential expression (proteome) analysis in the whole blood in the PSY cohort. LogFC was calculated as $log2( \frac{{mean(2}^{cases})}{{mean(2}^{controls})}$). Beta indicates a corresponding 𝛽 coefficient in the linear model where the base level is represented by controls. P-value shows nominal significant two-tailed p-value from linear regression (function lm in R). Adj.P.val shows adjusted p-values with Bonferroni correction.

**Table S6.** This table shows results for differential expression (proteome) analysis in the whole blood in the PSY cohort adjusted for the antidepressant intake. The analysis was performed as a "complete case". LogFC was calculated as $log2( \frac{{mean(2}^{cases})}{{mean(2}^{controls})}$). Beta indicates a corresponding 𝛽 coefficient in the linear model where the base level is represented by controls. P-value shows nominal significant two-tailed p-value from linear regression (function lm in R). Adj.P.val shows adjusted p-values with Bonferroni correction.

**Table S7.** This table shows results for differential expression analysis in the brain tissues in the cohort GSE539887. LogFC was calculated as $log2( \frac{{mean(2}^{cases})}{{mean(2}^{controls})}$). ID shows a corresponding probe ID in the Human Genome U133 Plus 2.0 Array. Beta indicates a corresponding 𝛽 coefficient in the linear model where the base level is represented by controls. P-value shows nominal significant two-tailed p-value from linear regression (function lm in R). Adj.P.val shows adjusted p-values with Bonferroni correction.

**Table S8.** This table shows results for differential expression analysis in the whole blood in the cohort GSE64930. LogFC was calculated as $log2( \frac{{mean(2}^{cases})}{{mean(2}^{controls})}$). ID shows a corresponding probe ID in the Illumina HumanHT-12 Array. Beta indicates a corresponding 𝛽 coefficient in the linear model where the base level is represented by controls. P-value shows nominal significant two-tailed p-value from linear regression (function lm in R). Adj.P.val shows adjusted p-values with Bonferroni correction.

**Table S9.** This table shows results for differential expression analysis in the whole blood in the cohort GSE98793. LogFC was calculated as $log2( \frac{{mean(2}^{cases})}{{mean(2}^{controls})}$). ID shows a corresponding probe ID in the Human Genome U133 Plus 2.0 Array. Beta indicates a corresponding 𝛽 coefficient in the linear model where the base level is represented by controls. P-value shows nominal significant two-tailed p-value from linear regression (function lm in R). Adj.P.val shows adjusted p-values with Bonferroni correction.

**Table S10.** This table shows results for differential expression analysis in the whole blood in the cohort GSE46743. LogFC was calculated as $log2( \frac{{mean(2}^{cases})}{{mean(2}^{controls})}$). ID shows a corresponding probe ID in the Illumina HumanHT-12 Array. Beta indicates a corresponding 𝛽 coefficient in the linear model where the base level is represented by controls. P-value shows nominal significant two-tailed p-value from linear regression (function lm in R). Adj.P.val shows adjusted p-values with Bonferroni correction.
